# Early neural encoding of pitch drives cue weighting during speech perception

**DOI:** 10.1162/IMAG.a.1082

**Published:** 2026-01-06

**Authors:** Magdalena Kachlicka, Ashley Symons, Fred Dick, Kazuya Saito, Adam Tierney

**Affiliations:** School of Psychological Sciences, Birkbeck College, University of London, London, United Kingdom; Department of Psychology, Royal Holloway, University of London, London, United Kingdom; Department of Experimental Psychology, University College London, London, United Kingdom; Department of Culture, Communication & Media, Institute of Education, University College London, London, United Kingdom

**Keywords:** speech, pitch, EEG, prosody, auditory

## Abstract

Linguistic categories are conveyed in speech by several acoustic cues simultaneously, so listeners need to decide how to prioritize different potential sources of information. There are robust, replicable individual differences in how cues are weighted during speech perception, but the underlying mechanisms explaining this variability remain unclear. Here, we tested the hypothesis that the reliability of early auditory encoding of a dimension is linked to the weighting placed on that dimension during speech categorization. This hypothesis was tested in 60 first language speakers of Mandarin-learning English as a second language. Neural tracking of changes in the pitch contour of naturalistic speech was assessed using EEG, while speech cue weighting was behaviorally tested using word emphasis, lexical stress, and phrase boundary categorization tasks. We find that neural tracking of pitch is linked to pitch cue weighting during word emphasis and lexical stress perception. Specifically, higher pitch weighting is linked to increased tracking of pitch at early latencies within the neural response, from 15 to 55 ms. These results suggest that individuals’ perceptual strategies are shaped by the reliability of encoding at early stages of the auditory system.

## 1 Introduction

Many biological and communication systems, including the immune system, the nervous system, and the genetic code, feature degeneracy: the same function can be carried out in different ways (Edelman & Gally, 2001). Degeneracy is distinct from redundancy, in which the same function is carried out the same way across multiple identical structures (Mason et al., 2015). One advantage of degeneracy is that it can make systems robust to disruption. The brain, for example, is a degenerate system, with multiple different structures capable of carrying out a single process (Friston & Price, 2003); as a result, functions can be preserved despite damage to single brain areas (Noppeney et al., 2004; Price & Friston, 2002).

Speech is another degenerate system: it contains multiple acoustic channels conveying the same information in different ways (Winter, 2014). The difference between an initial voiced versus unvoiced consonant in English, for example (as in “bat” vs. “pat”), is conveyed by as many as 16 different cues, including the time that elapses between consonant release and pitch onset and the fundamental frequency of the following vowel (Lisker, 1986). Similarly, word emphasis is conveyed by both lengthening of the word and a rise in pitch (Breen et al., 2010). Just as the degeneracy of the human brain makes it robust to damage, the degeneracy of speech enables it to be understood even in less-than-ideal conditions (Monaghan, 2017). For example, when background noise obscures temporal cues, listeners can instead rely on spectral cues to distinguish speech contrasts (Winn et al., 2013). Degeneracy also ensures that speech is robust to cross-speaker variability in production: although individual speakers vary in the extent to which they use spectral versus temporal cues when conveying linguistic categories such as word emphasis (Mousikou et al., 2024), this variation does not compromise the comprehensibility of speech (Ip & Cutler, 2022). Moreover, speech perception is robust to individual differences in auditory perception. Individuals with a severe difficulty with pitch perception, for example, report no difficulty with speech perception in everyday life (Liu et al., 2010) and can perceive speech prosody as well as the general population as long as alternate cues besides pitch are present in the signal (Jasmin et al., 2020a).

The degeneracy of speech makes it robust to distortion, but it also poses a problem for the listener: how should different acoustic channels be prioritized? In natural speech, acoustic cues often conflict, suggesting contrasting interpretations and requiring the listener to decide which cue should be discarded (Mousikou et al., 2024). Theories of speech perception suggest that cues are weighted by the reliability with which they signal linguistic categories in a given context (Kleinschmidt & Jaeger, 2015; Toscano & McMurray, 2010). On an implementational level, neural populations which code for acoustic characteristics that reliably signal the presence of linguistic categories could come to have a greater influence on downstream neural processes which detect those categories (Martin, 2016). During audiovisual speech perception, for example, the connectivity of the auditory and visual cortices with the superior temporal sulcus is driven by the relative reliability of the auditory and visual modalities across conditions (Nath & Beauchamp, 2011). Reliability-weighted connectivity could explain cue integration not only during speech perception but also during other perceptual tasks. For example, during visual-tactile integration (Beauchamp et al., 2010) and audiovisual spatial localization (Rohe & Noppeney, 2016, 2018), signals from primary sensory areas are integrated and weighted by their reliability in parietal cortex. Similarly, to carry out perception of heading during navigation, neurons in the dorsal medial superior temporal area weight visual and vestibular inputs by their reliability (Fetsch et al., 2012; Gu et al., 2008; Morgan et al., 2008).

If the relative weighting of cues is set by the distributional characteristics of the signal, then perceptual strategies should be similar across listeners. However, there are large individual differences in cue weighting across listeners, for first language (Choi, 2022; Hazan & Rosen, 1991; Mousikou et al., 2024) and second language (Kim et al., 2018; Schertz et al., 2015) speech perception. This variability replicates over time (Kim et al., 2018; Idemaru et al., 2012; Schertz et al., 2015) and correlates across tasks (Clayards, 2018; Kong & Edwards, 2015), suggesting that it reflects stable individual differences in perceptual strategies rather than inconsistency in responding. Variability in cue weighting can have consequences for language learning success, as certain strategies are better suited for learning speech sound contrasts in a particular language than others (Chandrasekaran et al., 2016; Iverson et al., 2003). Understanding why individuals weight cues differently, therefore, could lead to training programs designed to shift perceptual strategies. In addition, variability in cue weighting can be used as a window to explore the potential underlying neural mechanisms which carry out perceptual strategies.

What neural mechanism could explain variability in cue weighting? One possibility is that individuals differ in the reliability with which early auditory regions track acoustic characteristics of speech. This variability in sensory reliability could then lead to inter-individual differences in the connectivity between early auditory areas and downstream cortical areas which integrate input from multiple sensory regions. There is some preliminary support for this model. For example, individuals with congenital amusia, a severe domain-general difficulty with pitch perception, down-weight pitch during speech prosody perception (Jasmin et al., 2020a). This down-weighting is linked to a decrease in functional connectivity between pitch-sensitive regions in auditory cortex and left frontal cortex (Jasmin et al., 2020b).

One way to test the sensory reliability model of cue weighting is to investigate whether perceptual strategies are linked to early auditory encoding of the dynamics of acoustic dimensions in naturalistic speech. Here, we asked whether weighting of pitch as a cue to speech prosody categorization is linked to the reliability with which pitch is encoded in early auditory cortex in first language Mandarin speakers learning English as a second language. Categorization of three different prosodic features was assessed: word emphasis (Breen et al., 2010; Ip & Cutler, 2022), stress (Chrabaszcz et al., 2014; Fear et al., 1995; Mattys, 2000), and phrase boundary (de Pijper & Sanderman, 1994; Streeter, 1978). Although pitch is generally a stronger cue to word emphasis and stress, while duration is generally a stronger cue to phrase boundary, both dimensions have been shown to be used by listeners to perceive all three features. We predicted, therefore, that higher behavioral pitch weighting would be linked to more reliable neural tracking of pitch across all three features. Pitch encoding was assessed by measuring neural tracking of the pitch of naturalistic, continuous speech using EEG (Brodbeck & Simon, 2022; Teoh et al., 2019).

## 2 Methods

### 2.1 Participants

Seventy-five L1 Mandarin-speakers learning English as a second language were recruited from the greater London area. To ensure that participants were complying during the categorization tasks, we only included participants in data analysis who had a significant relationship between at least one acoustic dimension and their response patterns for each task (see below for details). Sixty participants (53 female) passed this threshold; the remainder were not analyzed. (These participants were the same dataset described in Kachlicka et al. (2024); note also that these exclusionary criteria were applied before analyzing the relationship between behavior and EEG). These participants had a mean age of 22.6 years (SD = 3.3) and had been resident in English-speaking countries, including the United Kingdom, for an average of 7.4 (3.2) months, with a range from 1 to 17 months. Participants’ English proficiency was assessed using the Lextale vocabulary test (Lemhöfer & Broersma, 2012); their performance ranged from 46.7% to 96.7% (M = 71.4%, SD = 10.8%). Participants reported an average of 5.0 (5.2) years of musical training. Written informed consent was obtained from all participants for being included in the study. Study procedures were approved by the ethics committee of the Birkbeck School of Psychological Sciences.

### 2.2 Speech categorization tests

Participants were tested on their use of two different acoustic dimensions—pitch and duration—as cues to three prosodic features: word emphasis, lexical stress, and phrase boundary.

### 2.3 Categorization stimuli

Speech categorization stimuli were taken from the MBOPP corpus of prosodic features in English (Jasmin et al., 2022). These stimuli were created by recording a single talker reading aloud a pair of sentences for each prosodic feature. Each of these sentences contained a passage that was identical lexically across the two recordings but differed in the location of a prosodic feature. For word emphasis, the full sentences were “Dave likes to STUDY music, but he doesn’t like to PLAY music” and “Dave likes to study MUSIC, but he doesn’t like to study HISTORY”. (The all-caps words indicate the location of contrastive focus, a type of word emphasis which highlights two words with different meanings.) For lexical stress, the recordings included single words “COMpound” and “comPOUND”, with the all-caps sections indicating the position of the primary stress of the word. For phase boundary, the sentences were “If Barbara gives up, the ship will be plundered” and “If Barbara gives up the ship, it will be plundered”.

To generate the stimuli, we isolated the lexically identical passages across both recordings. For word emphasis, the two target passages were “STUDY music” versus “study MUSIC”; for lexical stress, they were “COMpound” and “comPOUND”; and for phase boundary, they were “If Barbara gives up, the ship” and “If Barbara gives up the ship,”. These two versions of each passage were then morphed onto one another using STRAIGHT (Kawahara & Irino, 2005), a Matlab software package that can be used to create a synthesized speech stimulus by aligning two recordings along selected acoustic dimensions. We created a 4 × 4 stimulus grid for each prosodic feature by controlling the extent to which pitch and duration resembled either the early or late (emphasis/stress/boundary) recordings. Specifically, pitch and duration values were set at 0% (identical to the early recording), 33%, 67%, or 100% (identical to the late recording).

### 2.4 Categorization procedure

Each of the three categorization tests began with an explanation of the linguistic feature being categorized and a presentation of a pair of stimuli from the unambiguous corners of the stimulus space (0% pitch/duration and 100% pitch/duration). Participants then completed two practice trials in which they were presented again with these unambiguous stimuli and asked to categorize them as early or late emphasis/stress/boundary by pressing one of two buttons on the screen. These buttons were labeled “STUDY music” and “study MUSIC”, “COMpound” and “comPOUND”, and “If Barbara gives up, the ship” and “If Barbara gives up the ship,” for the emphasis, stress, and boundary tests, respectively. Participants needed to answer both questions correctly to move on to the main test; if they failed to do so they completed these two practice trials again.

During the main test, the participants completed 10 blocks of 16 trials for each of the three prosodic features, for a total of 160 trials per feature. (Participants also completed 160 trials of a musical beat categorization test, which is described and analyzed in Kachlicka et al. (2024) but will not be analyzed here.) Each block contained a random ordering of the 16 possible stimuli for a single feature, selected without replacement. Because these tasks were unavoidably repetitive, to reduce boredom, categorization blocks were interleaved across features in the following order: musical beats, word emphasis, lexical stress, and phrase boundary. During each trial participants were presented with a single stimulus and were asked to press one of two buttons to indicate whether they perceived it to be early versus late emphasis/stress/boundary.

### 2.5 Data processing

To assess the extent to which participants used pitch versus duration information when deciding how to classify each stimulus, we used Firth’s bias-reduced logistic regression (package logistf in R) to assess the relationship between pitch and duration levels and responses on each trial. Pitch and duration levels were encoded as 1 (0% morphing level) through 4 (100% morphing level), while responses were encoded as 0 (early emphasis/stress/boundary) and 1 (late emphasis/stress/boundary). Firth’s bias-reduced logistic regression was used to minimize the problem of inflated coefficients when there are sharp perceptual boundaries in the stimulus space (Heinze & Schemper, 2002). A separate regression was run for each participant, and the coefficients for pitch and duration levels indicated how strongly these dimensions were weighted as cues to each prosodic feature. As noted above, to ensure that each participant successfully completed the task, we analyzed only data from participants for whom there was a significant relationship (p < 0.05) between at least one acoustic dimension (pitch or duration) and responses on each of the three tasks. Participants without any significant relationship between acoustic dimensions and responses were assumed to be responding randomly. Data from 15 participants were removed on this basis, leaving 60 remaining participants. The motivation behind this choice is that we wanted to ensure that our results were driven by differences across participants in perceptual strategies, as opposed to differences in task compliance. We have used this same exclusion criteria across several studies of cue weighting (Kachlicka et al., 2024; Symons & Tierney, 2024; Symons et al., 2024). In addition, we removed two anomalous outlying data points: one participant had a significantly negative cue weight for emphasis categorization, and another participant had a significantly negative weight for stress categorization. Both data points were extreme outliers (3.9 and 4.4 standard deviations away from the mean, respectively) and suggested potential misunderstanding of the task or the interface. The other data points from these participants were retained. (All results reported here remain significant if these data points are included.)

### 2.6 EEG stimuli

Stimuli for the EEG experiment consisted of a recording of the Ernest Hemingway story “The Old Man and the Sea” spoken by a single male talker. This stimulus has been used in previous studies of neural tracking of the pitch of speech (Teoh et al., 2019). Participants heard 10 segments of speech, with a mean duration of 180.8 seconds (standard deviation 10.3). After each segment was presented, there was a 1-second pause, followed by presentation of the next segment. Participants were not given a specific task but were simply asked to listen to the speech. The stimuli were presented through 3M EARtone ER-3A insert headphones at a volume of 80 dB SPL. Sound volume was calibrated using a sound level meter before every testing session.

### 2.7 EEG recording

EEG data were recorded using a Biosemi ActiveTwo system with 32 electrodes embedded in a fitted cap arranged according to the 10–20 system. Additional electrodes were placed at the earlobes for offline reference, with CML and DRL electrodes functioning as ground. Electrode offset was maintained below 20 mV. Data were recorded at a sample rate of 16,384 Hz and digitized with 24-bit resolution. The EEG was time-locked to the stimulus presentation by sending triggers just before the onset of each speech passage. An RTBox (Li et al., 2010) sent the triggers and detected the timing of clicks embedded at the onset of each stimulus, enabling the stimulus and EEG timing to be precisely aligned during data processing.

### 2.8 EEG data processing

FieldTrip (Oostenveld et al., 2011) was used for data pre-processing. Data were downsampled to 128 Hz and re-referenced to the average of the electrodes placed on the earlobes. Next, data were filtered using Butterworth filters; first, a low-pass filter with a cutoff of 30 Hz and a filter order of 6, and second, a high-pass filter with a cutoff of 0.2 Hz and a filter order of 4. These steps were all performed using ft_preprocessing. Filtering was done with a zero-phase forward and reverse filter. ft_componentanalysis was used to extract ICA components for artifact rejection; any component resembling eye blinks or eye movements was removed using ft_rejectcomponent. Data were then split into epochs aligned with the presentation of each of the 10 stimulus passages.

### 2.9 EEG data analysis

The multivariate temporal response function (mTRF; Crosse et al., 2016) was used to build a statistical model relating continuously varying characteristics of the speech stimuli to changes in the EEG at each channel. This technique uses regularized regression to predict the EEG potential at each time point based on stimulus characteristics within a range of preceding time points. The time points analyzed ranged from -50 to 350 ms; in other words, at a given time point in the EEG, the stimulus vector predictors were taken from between 350 ms before to 50 ms after the EEG. A doubly-nested procedure was used to tune the regularization parameter lambda, train the model, and test the model. First, nine of the trials were set aside for training, with the remaining trial reserved for testing. Leave-one-out cross-validation carried out across these nine trials (mTRFcrossval.m) was then used to determine the lambda that maximized the correlation between the predicted and actual EEG, with possible lambdas ranging from 10^0^ to 10^3^. The chosen lambda was then used to train the model across all nine trials and test the model on the remaining trial by comparing the predicted to the actual EEG using Pearson’s correlations. This process was then carried out 10 times, with each trial taking its turn as the testing trial. The r-values and model weights were averaged across all 10 iterations. The resulting r-values indicate the extent to which each stimulus characteristic is tracked by the EEG, while the model coefficients indicate the time course at which this tracking took place. During model training and testing the first 0.5 seconds of each stimulus presentation was removed to ensure that EEG responses did not primarily reflect stimulus onset.

The main predictor stimulus vector was the pitch contour of the stimulus. It was extracted using Praat at a sampling rate of 128 Hz and z-scored. In addition, to examine the specificity of the relationship between pitch tracking and cue weighting, we also extracted the amplitude envelope of the stimulus. This was done by first filtering the stimulus between 80 and 2800 Hz, taking the absolute value of the Hilbert transform of the result, low-pass filtering the envelope, and then downsampling it to 128 Hz. The pitch contour and amplitude envelope were supplied by the authors of Teoh et al. (2019). First, we trained a model to use the amplitude envelope to predict the EEG of the left-out trial. The predicted EEG was subtracted from the actual EEG; we then trained a second model to predict these residuals from the pitch contour.

### 2.10 Statistical analysis

For each participant, we calculated the nine channels with the highest r-values when comparing the predicted to the actual EEG signal. This choice of the nine most reliable channels followed a previous study from our lab on neural encoding of acoustic dimensions (Symons et al., 2021). R-values and model coefficients were then averaged across these channels and subsequent analysis focused on this cross-channel data. Spearman correlations were used to assess the relationship between neural tracking r-values and weighting of pitch and duration for each speech categorization task, corrected for multiple comparisons using false discovery rate (Benjamini & Hochberg, 1995). We followed up on any significant relationships between neural tracking and cue weights by assessing the time window at which neural tracking of stimulus characteristics was related to individual differences in perceptual strategies. This was done by correlating the model weight at each time point with the perceptual cue weight using Spearman correlations, then correcting for multiple comparisons across time points using cluster analysis (Maris & Oostenveld, 2008), with a threshold for cluster inclusion of p < 0.05, an alpha of 0.05, and 1000 iterations.

## 3 Results

**Fig. 1.**
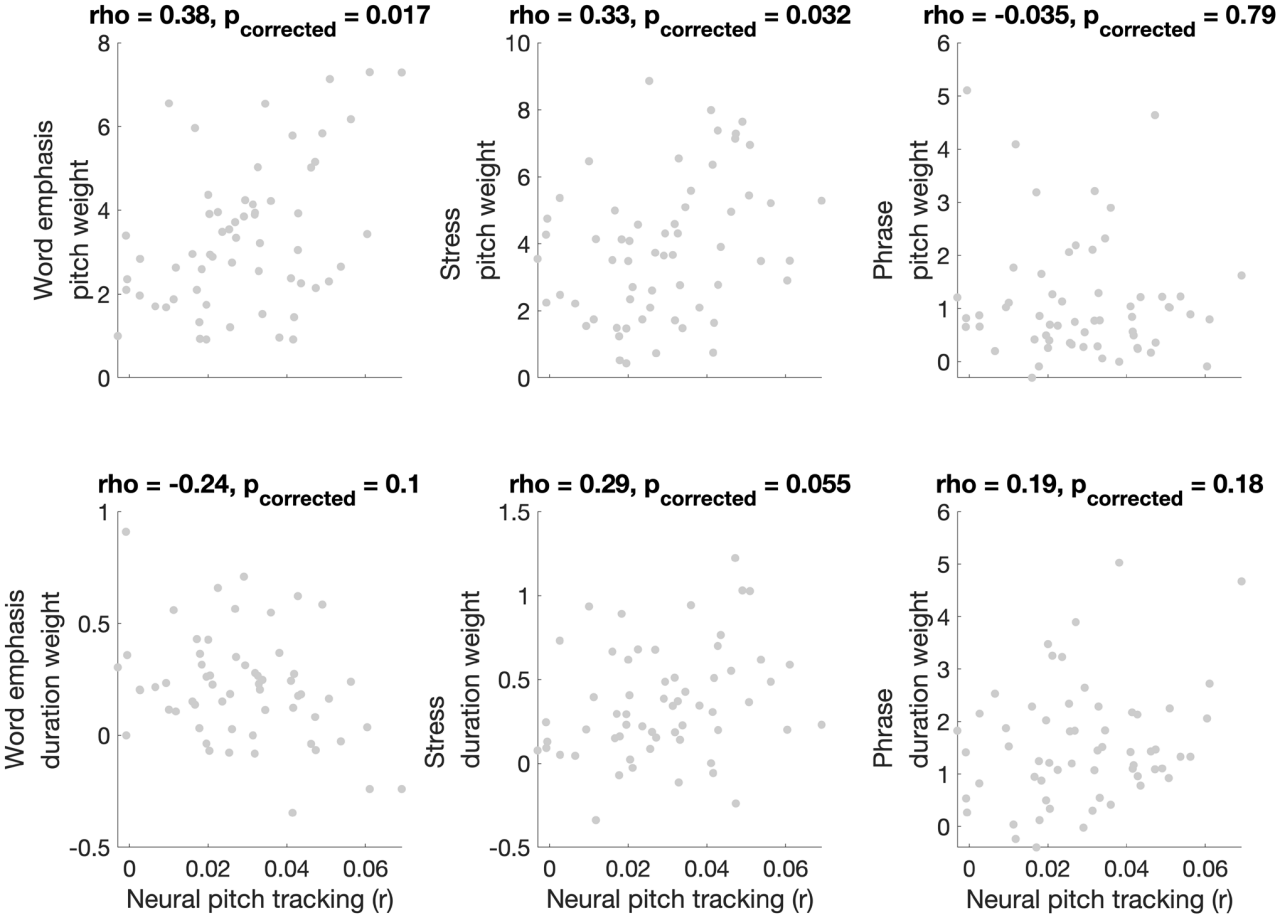
Scatterplots displaying the relationship between pitch tracking and pitch (top row) and duration (bottom row) weights during prosodic categorization. Prosodic features assessed included word emphasis (leftmost column), lexical stress (middle column), and phrase boundary (rightmost column). P values were corrected for multiple comparisons using false discovery rate (Benjamini & Hochberg, 1995).

**Fig. 2.**
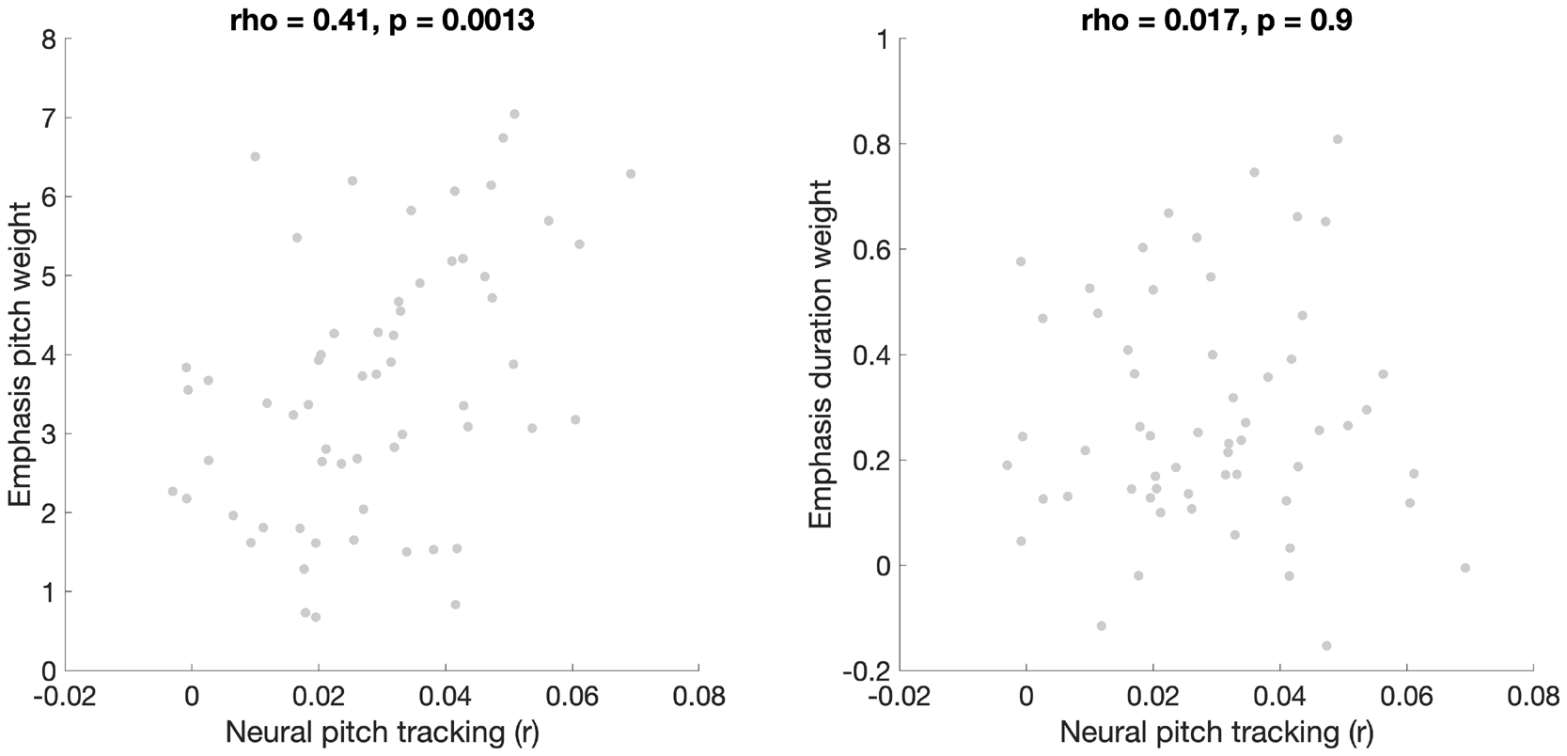
Scatterplots displaying the relationship between neural pitch tracking and pitch (left) and duration (right) cue weights, averaged across the two categorization tasks which involve perception of emphasis (word emphasis and lexical stress).

**Fig. 3.**
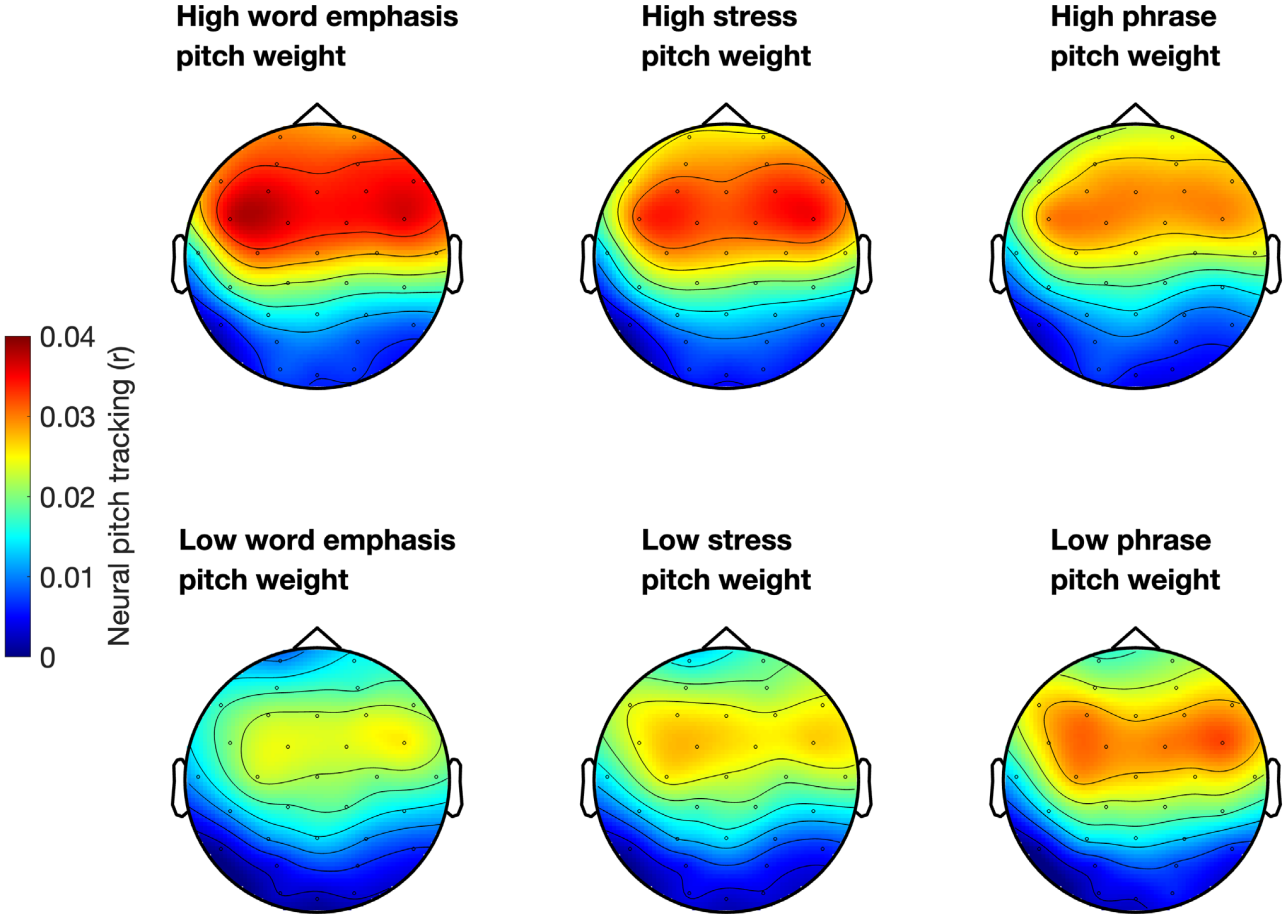
Topographical distribution of neural pitch tracking across the scalp in individuals with high (top) versus low (bottom) pitch weighting during word emphasis (left), lexical stress (middle), and phrase boundary (right) categorization. High versus low pitch weighting groups were defined as top versus bottom terciles.

We first examined whether neural tracking of pitch correlated with prosodic categorization cue weights. Scatterplots displaying the relationship between pitch tracking and pitch and duration weights across all three prosodic features are displayed in Figure 1. Neural pitch tracking correlated with pitch weights during word emphasis (rho = 0.38, p_corrected_ = 0.017) and lexical stress (rho = 0.33, p_corrected_ = 0.032) categorization. However, neural pitch tracking did not significantly correlate with pitch weights during phrase boundary categorization, nor with duration weights during any of the three categorization tasks (all p > 0.05). Given the similarity in patterns across the two tasks that involve categorization of emphasis (word emphasis and lexical stress), we next averaged cue weights across these two tasks (Fig. 2). Neural pitch tracking correlated with pitch weights (rho = 0.41, p = 0.0013) but not duration weights (rho = 0.017, p = 0.9) during emphasis categorization. Next, we used fisher’s r to z transformation (compare_correlation_coefficients.m; Ma, 2025) to compare these two correlations. Neural tracking of pitch was more strongly correlated with pitch weighting than with duration weighting (z = 2.21, p = 0.027). Figure 3 contains headplots showing neural pitch tracking across channels in participants with high versus low pitch weighting for each categorization task (top vs. bottom terciles). Neural tracking of pitch was frontocentrally distributed, as is commonly found in studies of neural tracking of speech (Teoh et al., 2019).

To further investigate whether the relationship with neural tracking of pitch is specific to cue weighting or whether it instead simply reflects attention to the task, we calculated performance on the unambiguous stimuli. We selected the most unambiguous portions of the stimulus space (the two stimuli in which pitch and duration both strongly indicate the same interpretation) and then assessed whether each of the participants’ 20 responses to these stimuli were correct or incorrect. The correlation between performance and pitch tracking was not significant for any of the individual prosodic features (all p > 0.05), suggesting that the link between neural tracking of pitch and pitch cue weighting was not driven by task attention or compliance effects.

**Fig. 4.**
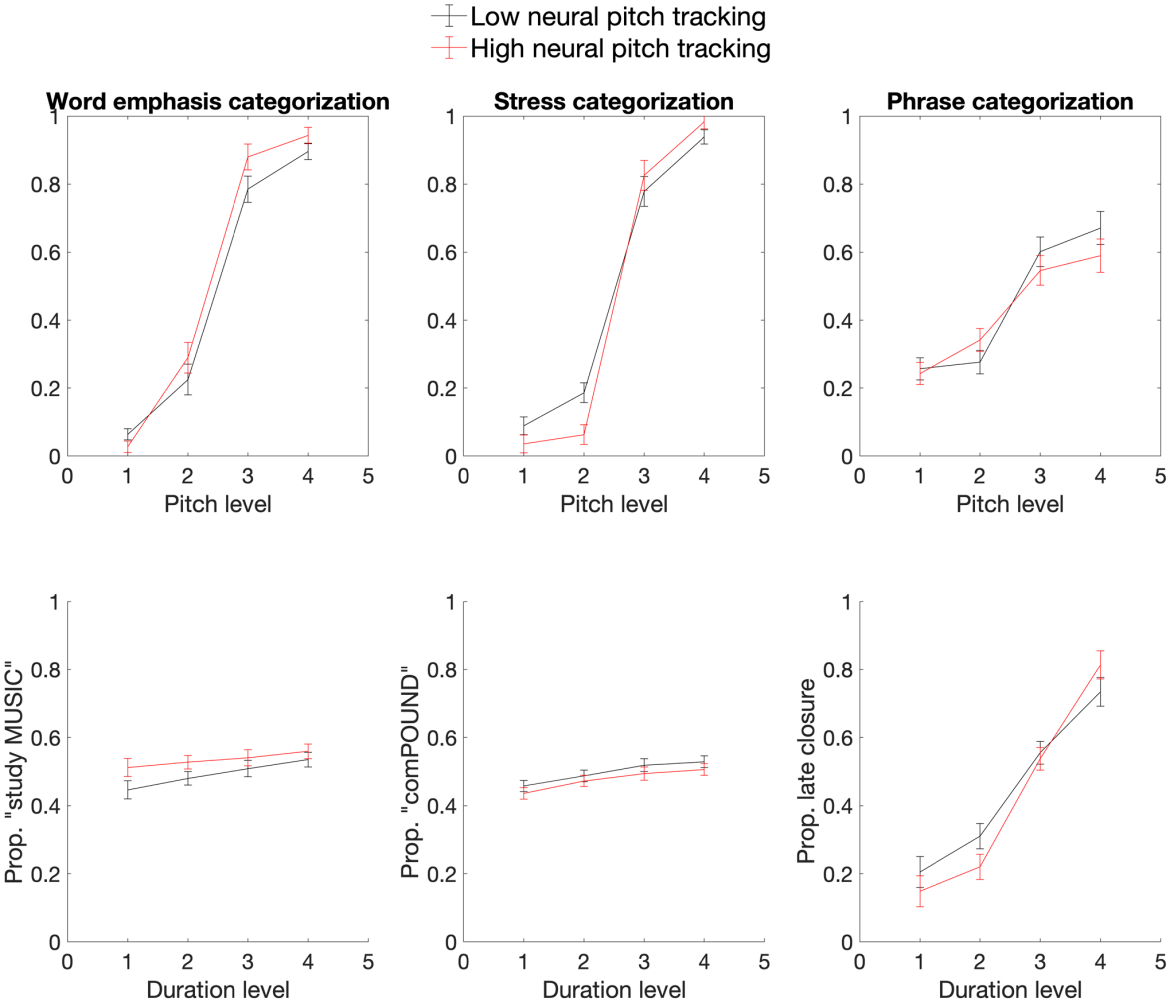
Line plots displaying how categorization responses changed as a function of pitch (top) and duration (bottom) stimulus morphing level in participants with high (red) versus low (black) neural tracking of pitch. High and low neural pitch tracking groups were defined as top versus bottom terciles, respectively. Error bars display one standard error of the mean.

To further illustrate the relationship between neural pitch tracking and categorization behavior, Figure 4 contains line plots showing how categorization changed as pitch and duration stimulus morphing levels were varied in participants with high and low neural pitch tracking (top vs. bottom terciles). For both word emphasis and lexical stress, the categorization functions were steeper in the high neural pitch tracking group, suggesting that these individuals made greater use of pitch when deciding whether they heard early or late emphasis/stress. There was, however, no clear group difference in categorization functions for pitch level during phrase boundary categorization, or for duration level during any of the categorization tasks.

Next, to determine the time window at which neural tracking of acoustic information is relevant for driving individual differences in perceptual strategies, we examined correlations between TRF weights and behavioral pitch weights for word emphasis and lexical stress categorization for each time point between 0 and 300 ms. (Weights at the edges of the time region included in the model—that is, -50 to 0 and 300 to 350 ms—were ignored to avoid the influence of artifactual edge effects, as recommended by Crosse et al., 2016.) This analysis focused on only pitch weights due to the lack of significant relationships between neural tracking of pitch and duration weights. Similarly, the analysis focused only on word emphasis and stress categorization due to the lack of a relationship between neural pitch tracking and phrase boundary categorization.

**Fig. 5.**
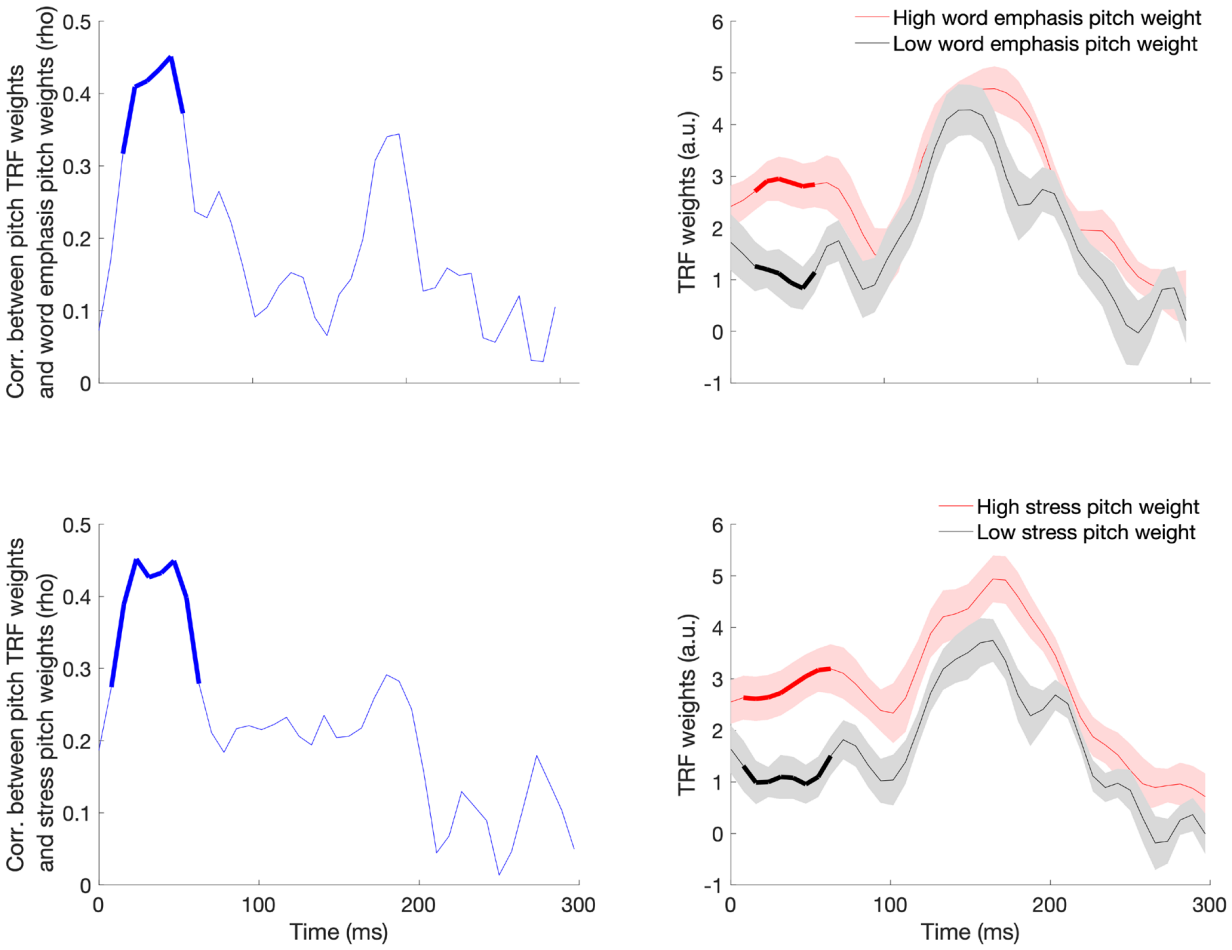
(Left) Relationship between pitch tracking mTRF weights across time and behavioral weighting of pitch as a cue to word emphasis (top) and lexical stress (bottom) categorization. The portions of the line marked in bold indicate the region which remained significant after cluster-based multiple comparison correction. (Right) Pitch tracking mTRF weights in high (red) versus low (black) behavioral pitch weighting groups for word emphasis (top) and lexical stress (bottom) categorization, defined as top versus bottom terciles. The shaded region depicts one standard error of the mean. The portions of the lines marked in bold indicate the time points in which the correlation between pitch TRF weights and behavioral pitch cue weighting survived cluster-based correction for multiple comparisons.

**Fig. 6.**
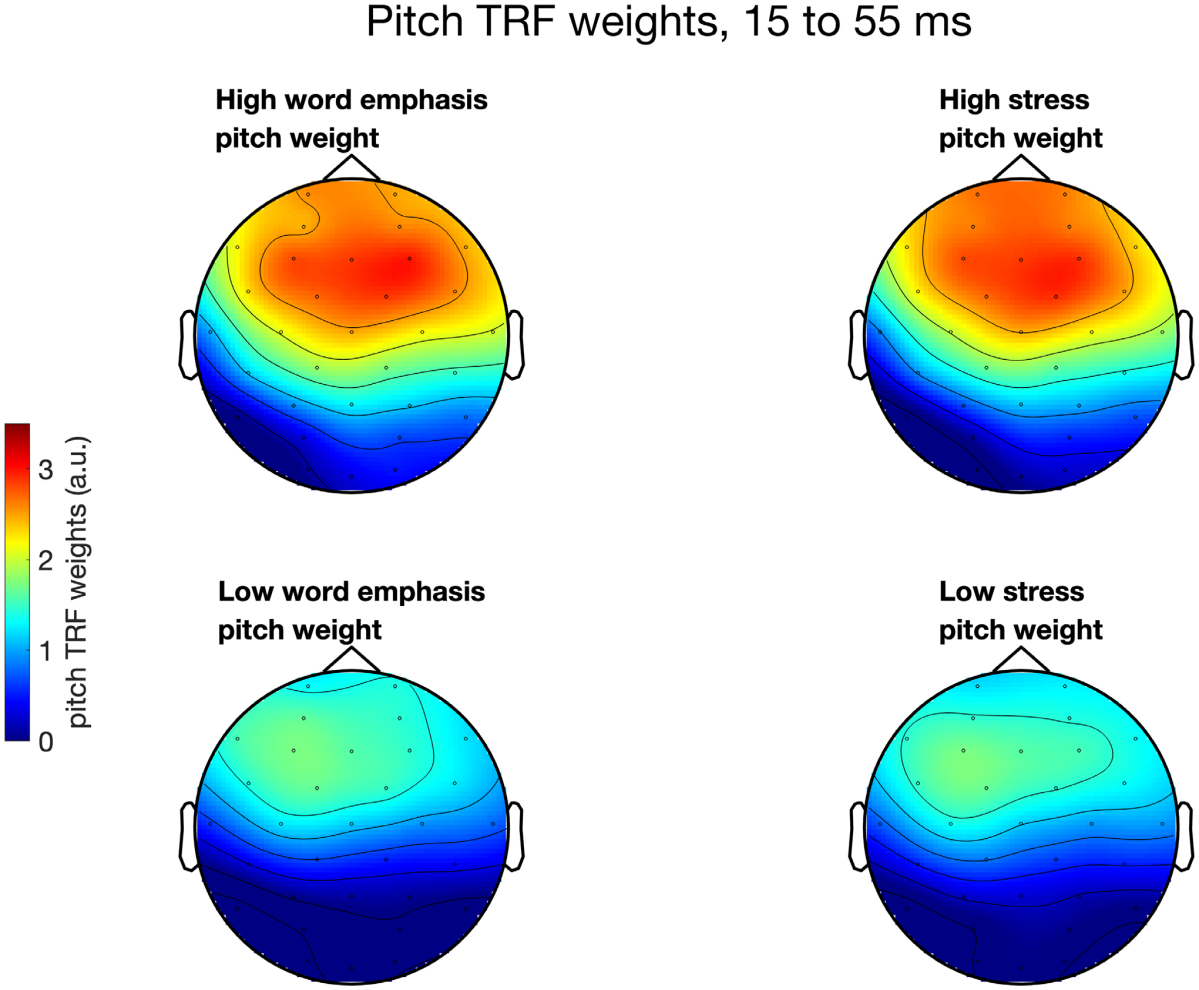
Topographical distribution of mTRF pitch tracking weights across the scalp, averaged across the time region 15 to 55 ms, in individuals with high (top) versus low (bottom) pitch weighting during word emphasis (left) and lexical stress (right) categorization. High versus low pitch weighting groups were defined as top versus bottom terciles.

**Fig. 7.**
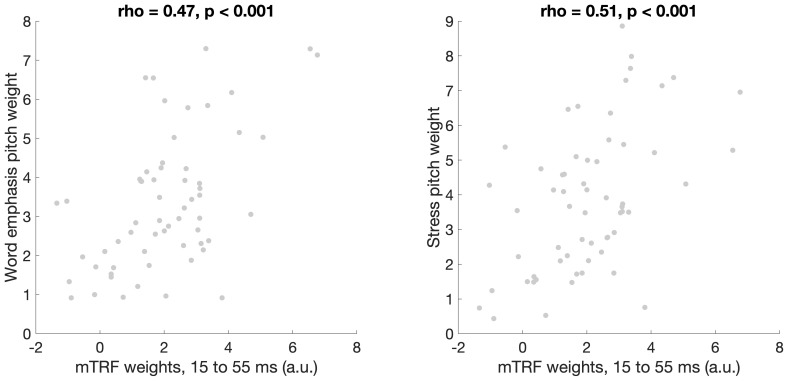
Scatterplots displaying the relationship between mTRF pitch tracking weights, averaged between 15 and 55 ms, and behavioral weighting of pitch as a cue to word emphasis (left) and lexical stress (right) categorization.

The correlations between mTRF weights and behavioral pitch cue weights displayed in Figure 5 were corrected for multiple comparisons using cluster correction. Figure 5 also shows the pattern of TRF weights in high and low behavioral pitch weighting groups for word emphasis and stress categorization. As can be seen in the righthand side of the figure, across all participants TRF weights peaked in two main regions, one with a maximum at around 70 ms and another at around 170 ms. This is consistent with the mTRF time courses reported by prior research on neural tracking of the pitch of speech (Brodbeck & Simon, 2022). Perceptual strategies, however, were specifically related to the earlier of these two windows: behavioral pitch weights for word emphasis and stress were linked to a cluster of mTRF weights across similar time windows (15.6 to 54.7 ms for word emphasis and 15.6 to 62.5 ms for stress), such that more pitch-biased perceptual strategies were linked to greater mTRF weights. Figure 6 shows the topographical distribution of mTRF weights during this time window in high versus low behavioral pitch weight groups for the word emphasis and lexical stress tasks (top vs. bottom terciles). Figure 7 shows scatterplots illustrating the relationship between the magnitude of mTRF weights in this time region and behavioral pitch weights during word emphasis (rho = 0.47, p < 0.001) and lexical stress (rho = 0.51, p < 0.001) categorization. Figure S2 shows pitch tracking mTRF weights across all 32 channels, averaged across all participants; the overall shape of the TRF is broadly similar across channels, and there is no apparent reversal of polarities across any pair of channels.

Finally, to further investigate the specificity of the relationship between pitch tracking and cue weighting, we ran a secondary analysis in which the amplitude envelope was first used to predict EEG dynamics. The predicted EEG was then subtracted from the actual EEG, and a second model was trained using relative pitch to predict these residuals. The resulting r-values were, again, correlated with pitch weights (rho = 0.27, p = 0.035) but not duration weights (rho = 0.054, p = 0.68) during emphasis categorization. Moreover, when correlating model weights from this residual analysis with pitch cue weights, we once again found that greater pitch weighting was linked to more positive coefficients in an early time period (23.4 to 54.7 ms for word emphasis, 15.6 to 62.5 ms for stress; see Supplementary Fig. S1).

## 4 Discussion

The degeneracy of speech affords multiple potential strategies to listeners during categorization. There are stable individual differences in which strategy listeners choose, and the neural mechanisms that guide this choice remain unclear. Here, we find that the reliability of early auditory neural encoding helps determine how strongly a given acoustic dimension is weighted during suprasegmental speech categorization. Specifically, we find that weighting of pitch as a cue to English speech prosody categorization is linked to neural tracking of pitch in L1 speakers of Mandarin. In particular, we find that it is neural tracking of pitch between 15 and 55 ms which is related to pitch weighting, with the correlation between pitch tracking and pitch weighting peaking at 31 ms. This latency range is consistent with a response arising from Heschl’s gyrus (HG), as electrocorticographic (ECoG) recordings have shown that responses in HG to speech range from around 25 ms in medial HG, 50 ms in middle HG, and 85 ms in lateral HG (Nourski et al., 2014). On the other hand, we find no significant link between cue weighting and neural pitch tracking at later latencies. Therefore, our results support a specific link between cue weighting and the reliability of neural representation of sound within primary auditory cortex, but not later auditory processing stages.

These results support a model in which cross-individual variability in the reliability of neural tracking of acoustic information in speech leads to individual differences in the connectivity between early auditory areas responsible for representing different acoustic dimensions and downstream cortical regions which integrate input across dimensions. This putative variability in connectivity could, then, drive individual differences in the weighting of cues during speech categorization. This model is also partially supported by recent findings from Ou et al. (2023), who reported that early neural encoding of the spectral and temporal cues relevant to categorization of a vowel contrast (as assessed by the frequency following response) was linked to relative weighting of these cues. Here, however, we extend the link between early auditory neural encoding and cue weighting to prosodic categorization and to a second language population. Thus, the role of the reliability of neural encoding of sound in speech cue weighting may not be limited to special cases but may instead by widely applicable to both segmental and suprasegmental categorization and to experienced and inexperienced language learners. Future research could test the hypothesis that the reliability of early auditory encoding is linked not only to behavioral cue weighting but to functional connectivity between early auditory areas and later areas which integrate across auditory cues. Some evidence in support of this hypothesis was provided by Jasmin et al. (2020b), who found decreased connectivity between pitch-sensitive regions within auditory cortex and left frontal regions in individuals with a severe deficit in pitch perception. However, it remains unclear whether this link is limited to individuals with an auditory deficit sufficiently severe to warrant a diagnosis, or whether it instead extends to the general population.

We interpret the link between pitch cue weighting and early neural tracking of pitch as reflecting reliability-weighted integration of auditory cues: individuals with less robust pitch tracking use pitch less during speech perception. An alternate explanation, however, is that down-weighting of pitch during speech perception leads to less robust neural representation of pitch via top-down feedback. This interpretation would align with prior findings from Helbig et al. (2012), who investigated visual-tactile integration and reported decreases in activation within visual cortex when tactile input was more reliable and decreases in activation within primary somatosensory cortex when visual input was more reliable. The reliability weighting and top-down feedback explanations are not necessarily mutually exclusive: imprecise early auditory encoding of a cue could lead to meta-knowledge about its unreliability, which could, in turn, cause processing of the cue to be inhibited. Future research could test the role of top-down feedback in driving early auditory encoding of acoustic cues by manipulating the perceived reliability of pitch using contextual cues (for example, by ensuring that pitch information always either aligns or misaligns with disambiguating information from text), then assessing changes in neural tracking of pitch.

Our finding that pitch cue weights were only linked to very early cortical tracking of pitch and were not linked to pitch tracking at later latencies conflicts somewhat with prior investigations of the links between cue weights and MMN responses, an event-related potential (ERP) with a latency of around 200 ms after stimulus onset. For example, Ylinen et al. (2010) found that training-induced shifts in the use of spectral cues to vowel recognition were linked to an increase in mismatch negativity (MMN) responses to spectral cues. Moreover, Zeng et al. (2022) found that the group difference in weighting of pitch, duration, and intensity as cues to stress categorization between English and Mandarin speakers was linked to cross-group differences in the relative size of MMN responses to pitch, duration, and intensity. One possible explanation for this discrepancy is that cue weighting may relate to the size of neural responses to violation of expectation, which we did not measure in this study. More robust early neural encoding of a particular acoustic dimension (such as pitch) may be linked to more precise predictions along that dimension and, therefore, greater neural responses to prediction violation. Future research could embed violations of expectations along specific acoustic dimensions, such as pitch and formant frequency, into otherwise naturalistic speech to investigate the relationships between the reliability of early auditory encoding, the magnitude of prediction violation responses, and acoustic cue weighting.

We find that the link between neural tracking of pitch and pitch cue weighting is present for word emphasis and lexical stress categorization but not phrase boundary categorization. Across individuals pitch tends to be a more useful cue than duration for word emphasis and lexical stress, while duration tends to be more useful for phrase boundary categorization (Kachlicka et al., 2024). It’s possible, therefore, that the influence of pitch on phrase boundary categorization may be relatively limited except in cases where pitch perception is exemplary (or duration perception is unusually poor). Another possible explanation is that English phrase boundary cue weighting has been shown to change with length of residence in first language Mandarin speakers, with longer residence linked to less use of pitch and greater use of duration (Petrova et al., 2023). The relative importance of language experience as a factor driving perceptual strategies for phrase boundary perception in this population may limit the influence of early auditory neural encoding.

The ideal way to test the neural reliability explanation for individual differences in cue weighting would be to simultaneously assess encoding of multiple acoustic dimensions in speech relevant to categorization. However, it is difficult to measure neural tracking of duration in naturalistic speech; compared to pitch it is a relatively abstract measure that can only be computed at the offset of segments, it is highly driven by confounding lexical factors, and it is unclear which hierarchical level would be most appropriate to track (i.e., duration of phonemes, syllables, words, etc.). Future work could assess cue weighting in suprasegmental perception using pairs of dimensions which both lend themselves to measurement of neural tracking in naturalistic stimuli. For example, encoding of formant shifts versus pitch could be assessed as cues to lexical stress categorization.

In summary, the reliability of early neural encoding of the pitch of speech is linked to the extent to which individuals rely on pitch as a cue to prosodic categorization. This suggests that individuals develop speech perception strategies which take into account the reliability of their neural representation of different aspects of sound. The degeneracy of speech ensures that, despite each individual’s idiosyncratic auditory strengths and weaknesses, there is an optimal solution that will allow them to successfully perceive speech.

## Supplementary Material

Supplementary Material

## Data Availability

Processed data and analysis scripts can be found at: https://osf.io/7w9ty/.
