# Supplementary material for: Early neural encoding of pitch drives cue weighting during speech perception
Source: Imaging Neurosci (Camb). 2026 Jan 6;4:IMAG.a.1082. doi: 10.1162/IMAG.a.1082 (PMC12776616; doi:10.1162/IMAG.a.1082)
Supplement: Supplementary Material [file IMAG.a.1082_supp.pdf]

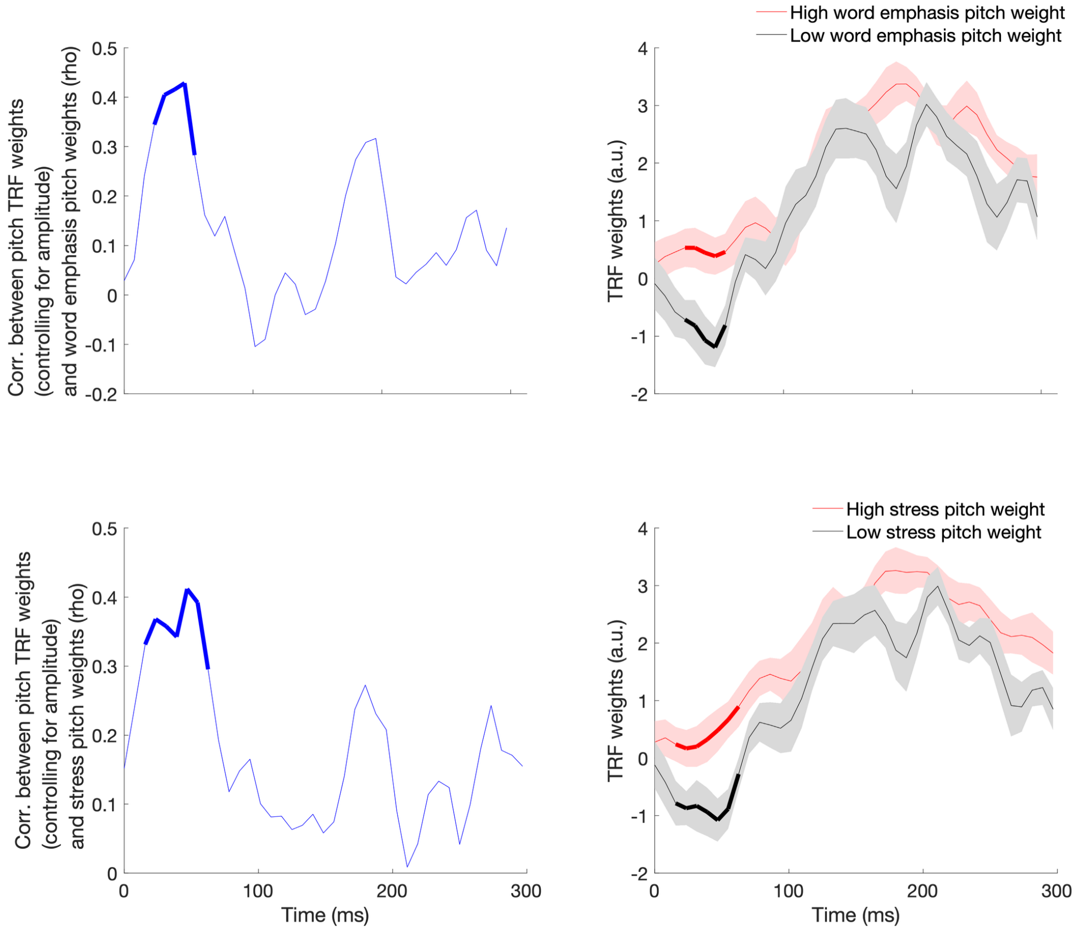

Figure S1. (Left) Relationship between pitch tracking mTRF weights (after controlling for amplitude envelope) across time and behavioral weighting of pitch as a cue to word emphasis (top) and lexical stress (bottom) categorization. The portions of the line marked in bold indicate the region which remained significant after cluster-based multiple comparison correction. (Right) Pitch tracking mTRF weights (controlling for amplitude) in high (red) versus low (black) behavioral pitch weighting groups for word emphasis (top) and lexical stress (bottom) categorization, defined as top versus bottom terciles. The shaded region depicts one standard error of the mean. The portions of the lines marked in bold indicate the time points in which the correlation between pitch TRF weights and behavioral pitch cue weighting survived cluster-based correction for multiple comparisons.

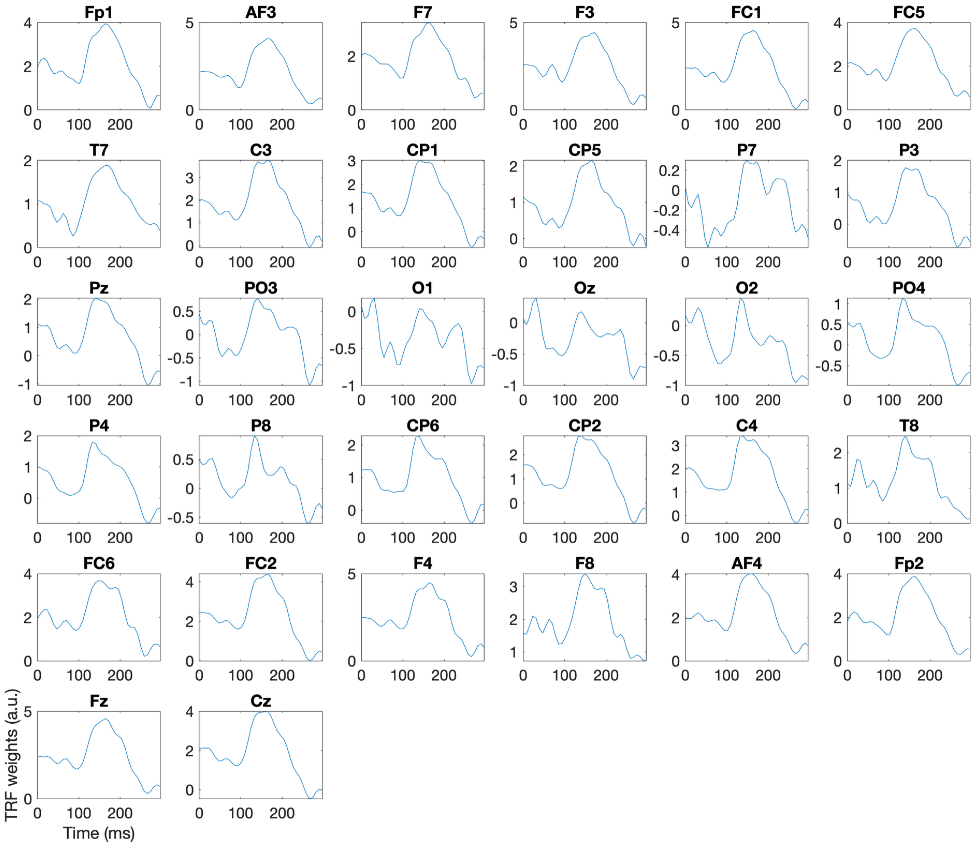

Figure S2. Pitch tracking mTRF weights across all 32 channels, averaged across all participants. The overall shape of the TRF is broadly similar across channels, and there is no apparent reversal of polarities across any pair of channels.
